# The Successful Treatment of Scarlet Fever; Also, Observations on the Pathology and Treatment of Crowing Inspiration in Infants

**Published:** 1859-03

**Authors:** 


ART. III.—*The Successful Treatment of Scarlet Fever; also Observations on the Pathology and Treatment of Crowing Inspiration in Infants.* By P. HOOD, Surgeon. 8vo., pp. 200. London: Churchill. 1857.

THIS little work consists of two distinct dissertations, embracing chiefly certain matters of fact which have fallen within the observation and experience of the author. The confident title of the first, and by far the longer of the two—"'The *Successful* Treatment of Scarlet Fever"—is well calculated to arrest the attention of every practical physician, for none of the diseases of childhood exhibit a greater mortality than this very exantheme, and none have given rise to a greater variety of opinion, not only as to its true nature, but also as to the best method of treating it. Moreover, either as a sporadic or epidemic disease, its geographical distribution is quite extensive. In his preface our author endeavors to anticipate any objections that might be urged against the propriety of such a title, by saying that it "only expresses what he has experienced to be true; and that the still longer experience of a friend—whose treatment of the disease is similar to his own, and based on the same principles—confirms what he has stated." Turning to page 6, we learn that during twenty-five years of active practice Mr. Hood has lost but two patients from scarlatina. One of the two cases—that of a boy seven years old—was moribund when Mr. Hood first saw it. The other was a girl four years old, in whom the fever and constitutional derangement being more than usually severe, he was prompted to adopt an antiphlogistic plan of treatment with diaphoretic, saline medicines, instead of his usual method.

He assures us that it was some years before he was able to reduce his mode of treatment to a state which was satisfactory to himself; that in the first fifteen years of his practice he frequently observed patients who, after passing through the primary disease favorably, were often prostrated and brought into extreme danger by some one or other of the various complications so commonly observed to follow scarlet fever,—such as pulmonary œdema, dropsy, anasarca, etc., and finally, that within the last ten years of his career as a practitioner he had learned to treat the disease so as to be able to prevent any of these complications from occurring. He informs us furthermore, that his friend Mr. Fuller, of Piccadilly, whose method of treating scarlatina closely resembles his own, has not lost a scarlet fever patient during a practice extending over thirty years. A statement similar to this may be found in Dr. Morris's work on scarlatina. A highly respectable physician informed Dr. Morris, in 1831, that he had never seen a fatal case of this disease, although he was extensively engaged in practice. We were present at a discussion on scarlet fever which took place at one of the meetings of the Philadelphia County Medical Society. One of the members, in the course of his remarks, said that he had attended many cases of scarlatina—more than 150, we believe—had treated them in the most simple manner, and had not lost one. This announcement called to his feet another member, who appeared scarcely able to find language sufficiently forcible to express his combined astonishment and incredulity. Upon further inquiry it appeared that these cases were all of a very light form—cases in fact of scarlatina simplex, which Watson thinks "is scarcely a disease," and which Sydenham long ago regarded as "fatal only through the officiousness of the doctor." In general, we see no positive necessity of calling the truthfulness of such statements into account. It is well known that in some epidemics the disease in question exhibits such a light and simple form as to require little or no treatment, almost every case recovering spontaneously. In other epidemics again, the fever assumes such a malignant character that no treatment, however judicious, or however timeously instituted, has the least control over the malady; and the mortality is frightful. There are epidemic cycles of the disease, requiring very long periods of time to complete their course, and exhibiting, from time to time, many minor and local changes in type. For these reasons it is very difficult to determine satisfactorily the value of any plan of treatment. In other words, it is not easy to satisfy ourselves whether the success which follows a certain kind of treatment is due to that treatment, or is simply at once the result and evidence of a strong tendency to health, inherent in the peculiar type subjected to such treatment. Before we can ascertain whether there has been any progress in the treatment of scarlet fever, we must first know whether the type of this disease has changed or not. We are inclined to think that such an inquiry would neither be so difficult nor so fruitless as at first sight it might seem. The differential diagnosis is so simple, and has been so well known since the publication of Withering's Essay, in 1779, that there can be little doubt as to the true character of the various scarlatinal epidemics which have occurred in different localities during the last eighty years. These epidemics were, in all probability, identical with the particular disease now known as scarlet fever. But their intensity or malignancy may have varied. It is obvious, therefore, that the intensity of an epidemic must be known before we can pronounce authoritatively upon the absolute merits of any special plan of treatment which may have been instituted during that epidemic. From a statement already made it will be seen that our author sets forth only the very general result of his twenty-five years of practice. He gives us no illustrative, individual facts, and we are left in total ignorance of the number of cases treated, and of the hygienic and social condition of his patients. We are not informed as to how many epidemics Mr. Hood witnessed in twenty-five years, nor how these epidemics differed from each other. These points, so important in their relations to the treatment, have been strangely overlooked.

Let us examine now the particular plan of treatment which in the hands of Mr. Hood has proved so eminently successful in scarlet fever, and which, we may say in passing, contrasts so strongly with that briefly set forth on page 1019 of the second volume of this journal. Before doing so, however, it may be well to premise that our author regards a peculiar blood-poisoning as the cause of the disease under consideration, and manifests a strong inclination to ascribe all the dangerous symptoms and complications to the accumulation of vitiated bile in the system.

The remedies which he employs are emetics, purgatives, and tonics. These are given, moreover, in the order in which they are here stated. When called to a case of scarlet fever, yet in its incipiency, he administers an emetic consisting of powdered ipecacuanha and sulphate of zinc. For a child six years old, he recommends the following formula: ℞.—Pulv. Ipecac. Rad., Zinci Sulphat. āā gr. x; Syrup, f℥j, and water, f℥j. This is to be followed by as much warm water as the child can drink.

"It will be observed, that however difficult the power of swallowing may be, however extensive the internal and external swelling, whatever amount of suffusion of the eyes, or injection of the face, all these symptoms will be diminished after vomiting is produced: the child breathes better, swallows with less difficulty, and a state of tranquillity succeeds to one of restlessness. The skin immediately becomes moister; the kidneys act freely, and the bowels are generally relieved. The child will frequently, on the subsidence of the emetic action, complain of being hungry, and ask for something to eat; but this desire should on no account be gratified, unless with a small quantity of the simplest article of food, such as bread, or biscuit. The more severe the form of the disease, the greater will be the relief afforded by this remedy, which is as applicable to malignant scarlet fever as to the milder varieties. No fear need be entertained of the continuance of vomiting, from the use of this form of emetic: the same, however, cannot be said of another which is a favorite with many, namely, tartar emetic. The action of this is much more uncertain, more violent, and more depressing in its after effects; therefore, I never use it."

If sudden swelling of the throat and difficulty of breathing come on at the close of the disease, especially during the periods of desquamation, recourse is again to be had to emetics as the best means of affording immediate relief. According to Mr. Hood this relief is not so great, however, as that noticed at the onset of the disease. With regard to the effects produced by emetics, when given under these circumstances, he thus speaks:—

"One of the most important effects produced by the exhibition of an emetic is that it thoroughly empties the gall-bladder of any acrid or redundant bile that may be in it, and at the same time clears the stomach of all such offensive matter as may either have entered it or have been secreted by it. Its effects upon the bowels are highly beneficial, owing to the increased quantity of bile which it causes to flow into them, and which operates on them as a stimulant. Its effect upon the kidneys in inducing a more copious secretion of urine is also most salutary in relieving the body through this channel; and the power which it exercises in relaxing the skin, causing a greater amount of perspiration to exude through its pores, is equally advantageous."

He employs emetics without hesitation in all convulsive cases also, unless there be positive evidence of a sanguineous congestion of the brain of an apoplectic character. The convulsions and coma which sometimes usher in an attack of the scarlatina "depend," according to our author, "entirely upon the presence of bile in the stomach; for when it has been discharged no repetition of these symptoms occurs, and no other kind of treatment that can be employed will act so speedily and satisfactorily in restoring the patient to perfect consciousness as an emetic."

In convulsive and comatose cases—

"The vitality of the brain has been so weakened by the presence of the scarlatinal poison in the blood which has been circulating through it, that after some indefinite period, depending on the constitution of the patient, and other circumstances, it becomes incapable of resisting any longer the morbid impression produced upon it by this vitiated secretion of bile, which has entered the stomach, and through the nerves of this latter organ, in communication with the brain, has occasioned the temporary suspension of its functions. If the mouth be opened and the tongue examined, it will be found loaded with a dark-brown fur and the saliva will be viscid and stringy. The impression conveyed on witnessing a patient in this state, is, that unless he be roused, or stimulated, death must soon ensue, for he seems, and he really is, on the verge of it already. Many are the remedies which I have known resorted to under such circumstances—brandy, ammonia, mustard poultices, blisters, bleeding, etc., but all generally without avail; unless the first-named, brandy, has acted as an emetic, which it sometimes will do, and then the object is attained which I contend for as being the only rational mode of affording relief, namely, vomiting. The administration of an emetic, under such circumstances, is *the* remedy; for until the stomach be emptied of the vitiated secretions contained in it, the brain will not return to a natural state, but every hour will increase the danger of effusion in that organ. If effusion has not taken place, the action of vomiting will speedily restore the patient to consciousness; but if effusion has occurred, owing to the length of time the patient has been permitted to remain comatose, or insensible, little more can be done to give him a chance of life. Mustard poultices, followed by blisters, and those remedies which are usually employed—but so frequently without success when effusion exists in the brain—may then be resorted to.

"If the emetic be long in acting, as it mostly is, in consequence of the diminished nervous energy of the stomach, a teaspoonful of pure brandy may be poured from time to time down the throat until this action is excited, and the fauces and back part of the throat may be tickled with a feather."

Our author also recommends the administration of emetics in cases approximating in character to the worst forms of typhoid fever, contending that as long as any bile or morbid secretions are present in the stomach, neither stimulants nor tonics will be of any avail, and the patient will derive no advantage from either, as their effects will be to increase the amount of oppression already existing in the system.

In the simple forms of scarlatina, when the action of the emetic has subsided, and in convulsive and comatose cases when sensibility has been restored, and the heart and pulse have regained their wonted vigor, and the skin has become warm, a brisk purgative is next to be administered. The particular purgative which Mr. Hood is in the habit of using, is "scammony and calomel." We suppose he alludes to the Pulv. Scammonii cum Calomelane—a preparation commonly in use among British practitioners, and consisting of five grains of scammony, two and a half grains of calomel, and two and a half grains of sugar, in every ten grains of the powder. Of this powder he gives six grains to a child five years old. This medicine he administers every night, not, however, in the same quantity, but in doses of three or four grains. When it has operated more than twice during the day, he omits it for a night. The use of this powder he continues until the fever has quite subsided, the tongue become perfectly clean, and desquamation has been thoroughly accomplished. To aid this latter process the warm-bath is to be employed every second night for a week or ten days, great care being taken that the child is not chilled on coming out of the bath. If the tongue becomes clean, and remains so after the operation of the emetic and the first dose of the aperient medicine, the powder of scammony and calomel is to be replaced by some of the simplest laxative medicines, such as rhubarb, castor oil, etc.

"I have never seen any ill effects arise from the administration of purgatives in scarlet fever, when given in the form and manner which I have described. I entertain the strongest conviction that if there be one form of medicine more than another which is entitled to preference, it is of this class. We are enabled by the use of aperients to do for nature what she is unable to perform for herself—that is, to discharge from the body those excrementitious matters, the retention of which is the grand cause of disordered action throughout the system. Aperients perform to a certain extent the work of the kidneys and skin, and by their means we can stimulate the bowels to throw off effete matters; but our power is limited, by causes previously mentioned, to make either the kidneys secrete sufficient urine to relieve the blood, or the skin to perform its proper function to answer the same object. The effect of aperients is to relieve from oppression all the vital organs; and, by a steady and persevering use of them, we need never be apprehensive of a critical diarrhœa, or a copious perspiration: all violent efforts of nature are spared the patient, and the progress to convalescence is rendered safe and easy.

"I regard, therefore, the judicious administration of purgatives as one of the most important and essential aids toward the successful treatment of scarlet fever. No complications of any kind are likely to occur if they be properly used, and given with discretion, according to the strength and constitution of the patient. The term "purgative" may, perhaps, have the effect of misleading some persons as to the particular effect to be derived from those medicines in the cases under consideration: but it is not a purging that is desirable; a moderately loose state of the bowels is all that is required by giving aperient medicine in scarlet fever, so as to insure a free discharge from the alimentary canal of all matters prejudicial to the body."

Having thus freely puked and purged his patient, our author next resorts to the sulphate of quinine in conjunction with dilute sulphuric acid and Huxham's tincture. To a child of six years he gives a grain and a half of the antiperiodic, with from eight to ten minims of the acid, and from half a drachm to one drachm of the tincture in one ounce of water sweetened with a drachm of orange-peel. To the use of quinine our author attaches great importance, as will be seen from the following quotations:—

"As I regard quinine to be the sheet-anchor of successful practice in scarlet fever, I am relieved of all anxiety as to the result of the disease, when I have once fairly established the regularity of its administration. * * *

"When proper attention is paid to the evacuation of the bowels, and the system by such means is relieved from all poisonous excrementitious matters, quinine, in combination with the dilute sulphuric acid, will then be found to produce its antiseptic and febrifuge effects in the most satisfactory manner. The accelerated action of the heart will abate, the skin will become cooler, and the nervous irritability—so strikingly displayed as the result of some poisonous influence pervading the system—will be tranquilized. The rash will soon fade—it seldom or ever lasts longer than three days—and the swelling of the tonsils will diminish, and gradually subside.

"No superficial ulceration will occur in these organs; the soft parts covered by mucous membrane within the mouth and throat will be observed to assume a healthier hue, and the swelling of the face and eyelids will soon subside. Conviction is thus forced on the mind that quinine is the antidote to the specific poison of scarlet fever, provided it be so administered as to enable it to exercise its most valuable properties in the way sought for. The curative efficacy, however, of quinine, like that of every other remedy applied through the stomach, depends upon the due absorption of it into the blood by the process of digestion: if it digests well, it soon enters the blood, rapidly puts a stop to its further deterioration, and thereby communicates the power—indirectly in some cases, positively in others—to each vital organ to perform its special function properly, and with benefit to the general system. This, however, it will not do unless the system, by the use of purgatives, as has been previously urged, is kept free from effete matters, which, otherwise, will most prejudicially interfere with its action. When this most important preliminary object has not been attained, quinine, instead of allaying the rapid action of the heart, accelerates it, at the same time rendering the skin hotter and the rash more vivid in color, increasing the swelling of the face and eyelids, and injuriously affecting the internal mucous membranes. The eyes acquire additional brilliancy, the patient complains of frontal headache, and his restlessness becomes painful to witness. If these symptoms are allowed to continue, delirium of an active kind will supervene, and one or other of the vital organs of the body will soon display some decided evidence of the exercise of its peculiar or appropriate function being interrupted."

Mr. Hood informs us that he has found quinine very beneficial in all those affections of the throat in which the glandular structures and mucous membranes are implicated, as in œdematous laryngitis and tracheitis, tonsillitis, cynanche parotidea and cyanche maligna. He advocates its use in erysipelas and diphtherite also.

"My experience of the treatment of erysipelas, in some of its worst forms, is exactly that which I have found most successful in the treatment of scarlet fever, in all its varieties: the same remedies, but usually in larger doses, as far as quinine is concerned, with the addition of wine and other remedies of a sustaining kind, bring this disease to a satisfactory termination."

Mr. Hood employs wine more frequently in cases of scarlet fever occurring among adults, than in those of young children. He prefers port wine, and gives it in quantities ranging from half a bottle to a bottle per day. Sound old wine is the best on account of the readiness with which it digests. New port wine is more difficult of digestion, and from the greater quantity of alcohol it contains, it is more stimulating and less tonic than old wine. Where thirst is excessive, and there is much irritability of stomach old hock is to be preferred to port wine.

"Whenever the use of wine is indicated by the state of the body, quinine is equally, if not more so. In the malignant sore-throat, with or without a rash, the prompt and decided administration of these two remedies may be said to constitute the basis of successful treatment.

"When the tonsils and soft parts at the back of the throat are of a mulberry color, sometimes approaching to a dark-grayish blue, with ash-colored sloughs, the danger from speedy exhaustion is imminent. I have, however, hitherto always found this form of disease perfectly under control by the free exhibition of wine and quinine.

"Should the medical attendant become irresolute, in consequence of the patient showing symptoms of delirium, and hesitate in having recourse to the aid of wine and quinine, lest they should promote a state of congestion in the brain, the peril of the patient will be increased.

"As this form of delirium is the result of an impure highly carbonized blood feebly circulating through the brain, the grand object ought to be the promotion of a more vigorous circulation, by stimulating the action of the heart; to establish a freer respiration, to enable the whole of the lungs to expand and receive air, so that the blood may be as speedily as possible divested of its poisonous element, by the natural action of breathing."

The extreme restlessness and insomnia which sometimes characterize scarlet fever, are to be subdued with opium. Mr. Hood thinks it unsafe to give this drug, if the bowels have not been properly emptied, or if the tongue be loaded with a yellow fur, indicative of the presence of bile in the stomach. He asserts that when given under these circumstances, it will still further complicate existing difficulties, and render the brain susceptible of the injurious effects of the sedative. "The proper time for prescribing opium is after the bowels have been freely emptied, and the tongue has become clean; and if that organ should have a preternatural red color, and seem as if it were glazed, and the patient be sleepless and restless, opium must be given, or low fever may ensue."

When the tonsils are ulcerated Mr. Hood touches them with the solid nitrate of silver. To relieve the dryness of the throat and difficulty of swallowing he uses a gargle made by adding the juice of one large lemon, and a tablespoonful of honey to a pint of *thick* barley-water, or rather barley-*gruel*. He is opposed to the abstraction of blood from the throat by leeches, to reduce the excessive swelling; but prefers, under these circumstances, the application of heat, by means of a hot flaxseed poultice put into a stocking, and placed as hot as can be borne around the throat.

The fourth and last chapter of this treatise is devoted to the consideration of the pathology and treatment of the dropsy which follows scarlet fever. The medicines which Mr. Hood has found most efficacious in subduing the acute form of this distressing and dangerous sequela are the bichloride of mercury, tincture of digitalis, and sweet spirits of nitre. To a child eight or ten years old he recommends the following draught, to be given every six hours, until the dropsical swelling abates: ℞.—Liq. hydrarg. bichloridi, ♏xv; Tinct. digitalis, ♏ij ad iij; Spirit. æther. nit. fʒss; Aquæ, f℥j. A powder composed of a grain to a grain and a half of calomel, with the same quantity of James's powder and sugar, is to be given every night, or every other night, as the case may require, followed in the morning by an effervescing, diuretic aperient draught, containing a drachm to a drachm and a half of tartrate of potash, half a drachm of bicarbonate of potash, half a drachm to a drachm of spirits of nutmeg; a drachm of syrup of ginger; and water an ounce and a half; to be taken with a tablespoonful of lemon-juice. In the mean time "the strength of the patient must be supported by a diet composed of milk and rice, and food of a farinaceous description; and where asses' milk can be obtained, it is of great service in sustaining the powers of the body when the circulation is rapid and the debility great. If there be much thirst, it may be quenched by raspberry vinegar and water, or the so-called 'imperial' drink may be used, which is a composition of cream of tartar, lemon-juice, sugar, and water." In the mild form of scarlatinal dropsy a "dose or two of calomel may be requisite at the commencement of the treatment; but the disease will generally be observed to yield readily to the administration of alteratives and chalybeates."

"I have seen," says Mr. Hood, "many cases in which the patients have rapidly got well by the use of medicine similar to the following: For children from six to eight years of age, hydrag. cum cretâ, a grain and a half; powdered squills half a grain; powdered jalap, four to five grains: to be taken every night. A mixture composed of tinct. ferri ammon., forty minims; syrup. aurantii, ʒiij; inf. cusparii, ℥iij; the half twice a day. In dropsies of this kind the nourishment must be liquid: it may consist of beef-tea, as well as milk, with the addition of rice and farinacea. A small quantity of wine and water may also be occasionally given with advantage."

In the treatise on Crowing Inspiration, our author records some original and valuable observations upon enlargement of the liver as a cause of this interesting and obscure disease. We regret that our limits, already exhausted, will prevent us from placing these observations before our readers.

